# Heavy Metals Pollution and Potential Health Risks: The Case of the Koche River, Tatek Industrial Zone, Burayu, Ethiopia

**DOI:** 10.1155/jt/9425206

**Published:** 2024-11-22

**Authors:** Mathewos Temesgen, Tegenu Alemu, Enkosa Shasho

**Affiliations:** Department of Biology, Ambo University, Ambo, Oromia Region, Ethiopia

**Keywords:** health risk, heavy metals, industrial waste, Koche River, pollution index

## Abstract

This study aimed to determine the levels of some heavy metals in the Koche River and the potential health risks. A replica of water samples was taken from 12 sampling sites purposely selected in the dry season. Heavy metal levels were determined using a flame atomic absorption spectrophotometer following the APHA (1998) procedure. Heavy metal pollution index (HPI), heavy metal evaluation index (HEI), chronic daily intake (CDI), hazard quotient (HQ), hazard index (HI), total hazard index (THI), and incremental lifetime cancer risk were calculated on the basis of the results. The heavy metals detected were Fe > Mn > Cu > Zn > Cr. The Cr, Fe, Mn, and Cu contents were above the maximum allowed limit of WHO for drinking and irrigation water at most of the sampling sites. The HPI and HEI values also surpassed the maximum limit of the study sites. The highest HPI and HEI values were found at the Yam1site. Oral ingestion represented 99.55% and 97.85% of CDI_total_ (CDI_ingestion_ + CDI_dermal_ contact) in adults and children, respectively. The mean CDI_total_ and the noncarcinogenic risk values were found in the order of Fe > Mn > Cu > Zn > Cr in both ages. CDI, HQ, HI, and THI scores were higher in children. The HI_oral_ and THI values were also higher than 1 in both ages except in DK 2, Sour 1, and Sour 2 sites. However, the HQ_dermal_ level was higher than 1 only for Cr in children. The ELCR obtained also indicated a high carcinogenic risk of Cr (0.75 ± 0.44 and 1.15 ± 0.66 in adults and children, respectively). In general, most of the study sites had heavy metal pollution levels that exceeded the maximum allowed limit. Therefore, effective management of sources of pollution and continuous monitoring of river quality to minimize health risks are very important.

## 1. Introduction

The rapid pace of industrialization, the increasing energy demand, and the reckless exploitation of natural resources in the last century have all contributed to the growing issue of environmental pollution, which threatens ecosystems and biological processes [1, 2]. Furthermore, rapid industrial growth and the discharge of polluted effluents into water bodies without adequate treatment are harming the environment [3]. Toxic metals such as arsenic, cadmium, copper, chromium, lead, mercury, nickel, and zinc are common heavy toxic heavy metals in polluted water [4]. They are widely distributed in the environment due to their numerous industrial, domestic, agricultural, medical, and technological applications, raising concerns about their potential effects on human health and the environment [5].

Heavy metals are one of the dangerous pollutants that cause havoc in our environment [2]. Due to their nonbiodegradability, long biological half-life, and ability to accumulate in aquatic ecosystems, they are among the most harmful water pollutants [6]. Heavy metal contamination of surface and groundwater resources has become a global problem due to its refractory properties and bioaccumulation [3]. The level of toxic effects depends on the metal, its biological role, and the organisms exposed to it [6, 7]. Furthermore, prolonged exposure to these metals above their permissible levels has negative consequences, including carcinogenic and noncarcinogenic risks such as neurological disorders and liver and kidney disease [8]. Therefore, constant monitoring of heavy metals in river water is critical for human health [9].

Several studies have been carried out on the pollution of rivers with heavy metals in Ethiopia, and the results show that it has a significant impact on the environment, plants, and aquatic animals in several ways [10]. Dietary exposure to heavy metals such as chromium, cadmium, lead, zinc, and copper has been recognized as a health risk for communities located near rivers that receive industrial waste [11, 12]. Asthma and allergies, lung cancer, eye disease, and cardiovascular problems, among others, are significant risks noticed in certain areas [13]. Furthermore, most of the rivers in and around Addis Ababa receive substantial amounts of pollutants from various industries without significant treatment [8, 14]. Riverside residents use river water for various purposes, such as washing clothes, cooking for livestock, domestic use, etc. Similarly, irrigation in the lower reach of rivers is heavily reliant on polluted rivers [15]. Therefore, investigating heavy metal levels in water is critical to understanding the current state of river water quality and threats to human health [16].

Burayu is a well-known city in Ethiopia due to its numerous industries, most of which are located in the Tatek Industrial Zone along the Koche River. These industries discharge their liquid waste into the river without adequate treatment, which poses a risk to farmers who live near the river. Despite extremely high pollution, there is no adequate water treatment system for the public water supply of the area, except for elimination of pathogenic organisms [8]. Farmers living along the banks of the Koche River use the river water for irrigation, livestock catering, and domestic consumption. However, there is no scientific information on heavy metal pollution in the river and its potential public health risks. Therefore, determining the level of pollution and the risk assessment is critical to assessing the quality of river water and the harmful effects of these pollutants. Therefore, this study aimed to assess the level of selected heavy metals (copper, iron, zinc, manganese, chlorium, cadmium, nickel, cobalt, and lead) in river water and the potential public health risks to the community living in the river area.

## 2. Materials and Methods

### 2.1. Description of the Study Area

**Figure 1:**
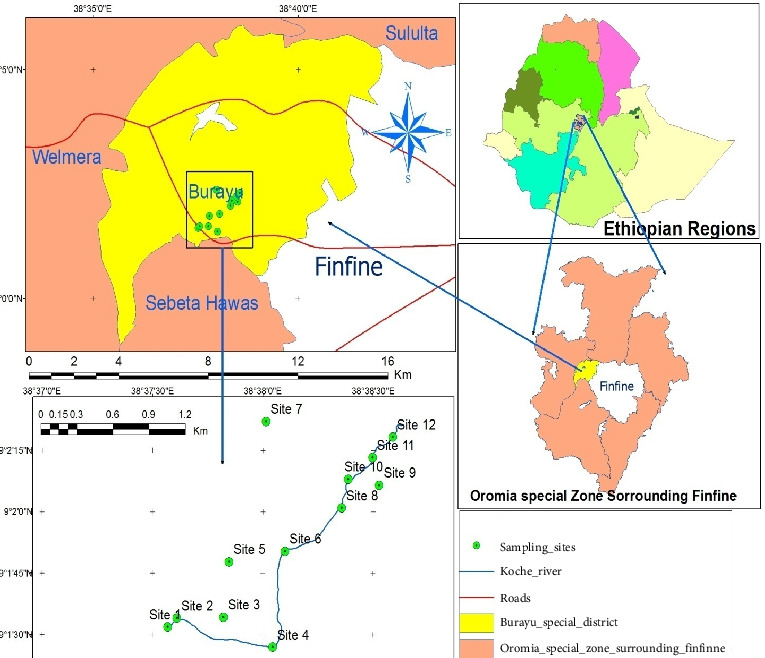
Map of the study area.

The study was carried out on the Koche River, which flows in the Tatek Industrial Zone, Burayu City, Oromia Regional State, Ethiopia, 14 km west of Addis Ababa in Gefersa Nono Kebeles (Figure 1). More than 202 large and medium industries, including marble, textiles, soap, plastics, paint, and other factories, are located in the industrial zone. The industrial zone has created employment opportunities for more than 17,320 people. People living near the river depend on mixed agriculture (farming and livestock production), which is dependent on river water. As a result, they are exposed to this river, which has positive and negative consequences.

### 2.2. Study Design, Data Types, and Sources

In this study, a cross-sectional experimental design that used a quantitative data collection approach was used for primary data collection. Water samples collected from various locations along the longitudinal gradient of the river were used as a data source.

### 2.3. Selection of Sample Sites

Before sampling, a preliminary observation was made in May 2021 in the study area to select the sampling site. Based on the information collected, 12 sampling sites were deliberately selected considering the level of pollution in the area. These include two sites in a source of the water at 100 m difference; four sites in the outlet of waste from garment, soap, marble, and food processing factories; four sites 20 m after each discharge joins the river; and two sites after all sources combined with the water (at 100 and 500 m) (Figure 1 and Table 1).

### 2.4. Water Sample Collection

Water samples were collected from the selected sites during the dry season (February–April 2023). Polyethylene bottles were washed with 10% nitric acid (HNO_3_) and distilled water, dried with a hot air blower, and rinsed with river water prior to sample collection. Thirty-six water samples (in triplication) were collected from the 12 selected sites using prewashed 1 L polyethylene plastic bottles. The samples were thoroughly mixed to create a composite sample and 1000 mL of water was collected from each location for laboratory analysis. All samples were labeled, placed in an ice box at 4°C, and brought to the Holeta Agricultural Research Institute laboratory for heavy metal analysis. As soon as the samples were brought to the laboratory, the samples were acidified using 2 mL of 2% HNO_3_ to avoid metal precipitation and microbial activity, filtered, and stored in the dark at ambient temperature until analysis.

### 2.5. Sample Digestion and Preparation

The water samples were digested according to the APHA [17] techniques. A glass fiber filter (GF/F) with a mesh size of 0.45 *μ*m was used to filter 50 mL of water sample. The samples were transferred to a block digester, and 10 mL of concentrated HNO_3_ was added and placed in the digester to evaporate to about 20 mL at 80°C. The heating continued and then a few portions of HNO_3_ were added until the solution appeared transparent. The solutions were filtered through Whatman filter paper No. 42, and the total volume was maintained at 50 mL with distilled water for heavy metal analysis.

### 2.6. Standard Preparation

All reagents used were analytical grade solutions. All working standards were prepared from certified standard solutions of 1000 mg/L. Acetylene gas was used as fuel and air as a support in the AAS Spectrophotometer, and an oxidizing flame was used in all cases except chromium, where a reducing nitrous oxide flame was used for metal analysis.

### 2.7. Analysis of Heavy Metals

Atomic absorption spectroscopy (AAS) (Agilent Technologies, 200 Series AA) was used for heavy metal analysis. The instrument was first calibrated using a calibration blank in triplicate. Standard solutions were diluted to the desired concentrations to calibrate the instrument. After being fitted with a specific lamp, the calibration curves for each heavy metal were set to ensure the accuracy and reliability of the instrument. Finally, the concentrations of heavy metals in the filtrates were analyzed [17].

### 2.8. Heavy Metal Pollution Index (HPI)

HPI helps estimate the combined general effect of individual heavy metal concentrations in water sources [2]. It was calculated based on the concentration of Cu, Mn, Zn, Fe, and Cr in the water using the values established by the WHO guidelines [18, 19] (equation (1)).

(1) $$\begin{array}{c} \text{HPI}=\frac{\overset{n}{\underset{i=1}{\sum }}=Q_{i}w_{i}}{\overset{n}{\underset{i=1}{\sum }}W_{i}}, \end{array}$$

where *Q*_*i*_ is a subindex, *W*_*i*_ is the unit weight (the ratio between *k* (*k* = 1) and the guideline values) of the *i*th parameter, and *n* is the total number of chemical elements considered (*n* = 7) [20]. *Q*_*i*_ was calculated using the following equation.

(2) $$\begin{array}{c} Q_{i}=\overset{n}{\underset{i=1}{\sum }}\frac{\left|C_{i}-I_{i}\right|}{S_{i}-I_{i}}*100, \end{array}$$

where *C*_*i*_, *I*_*i*_, and *S*_*i*_ are the concentrations of the *i*th element, the ideal, and the standard values of the WHO [21] guidelines for drinking water quality. Waters with HPI < 100 are considered unpolluted, whereas the HPI > 100 are classified as polluted and unsuitable for drinking [2]. The parameters used for the calculation of HPI are indicated in Table 2.

### 2.9. Heavy Metal Evaluation Index (HEI)

The HEI presents the overall water quality with respect to the heavy metal content in the water and is computed using the following equation [23]:

(3) $$\begin{array}{c} \text{HEI}=\overset{n}{\underset{i=0}{\sum }}\frac{H_{c}}{S_{i}}, \end{array}$$

where *H*_*c*_ is the determined value of the *i*th parameter, and *S*_*i*_ represents the guideline value of [18, 19] (Table 2). HEI classifies water as low (HEI < 1), medium (1 < HEI < 2), and highly polluted (HEI > 2) [23].

### 2.10. Human Health Risk Assessment

This helps to assess the levels of noncarcinogenic levels of heavy metal health risk through water consumption [24]. Participants in this study were divided into two age groups: children (0–20 years) and adults (21–70 years). The human health risk of oral and dermal exposure was determined. Chronic oral heavy metal intake (CDI_oral_) was calculated using the following equation [22]:

(4) $$\begin{array}{c} {\text{CDI}}_{\text{oral}}=\frac{\text{CW}*\text{IR}*\text{EF}*\text{ED}}{\text{BW}*\text{AT}}, \end{array}$$

where CW is the concentration of metal or ion concentration (*μ*g·L^−1^), IR is the ingestion rate (2.2 L·day^−1^ for adults/1 L·day^−1^ for children), EF is the frequency of exposure, ED is the duration of exposure, BW is the average body weight, and AT is the average exposure time [22, 25].

Similarly, CDI_dermal_ was computed as indicated in the following equation [22, 25]:

(5) $$\begin{array}{c} {\text{CDI}}_{\text{dermal}=}\frac{\text{CW}*\text{SA}*\text{Kp}*\text{ET}*\text{EF}*\text{ED}*\text{CF}}{\text{BW}*\text{AT}}, \end{array}$$

where CW is the concentration of metal or ion concentration (*μ*g·L^−1^), SA is the skin surface area, Kp is the coefficient of permeability, ET is the exposure time, EF is the exposure frequency, ED is the duration of exposure, CF is the conversion factor (1/1000 L·cm^−3^), BW is the average body weight, and AT is the average exposure time.

The input variables used to calculate the average daily dose of heavy metals by oral ingestion and dermal exposure are indicated in Tables 3 and 4.

#### 2.10.1. Hazard Quotient (HQ)

The HQ for oral ingestion and dermal exposure was calculated using equation (6) to assess noncarcinogenic health risks. HQ values greater than 1.0 indicate the presence of noncarcinogenic risk [22].

(6) $$\begin{array}{c} \text{HQ}=\frac{{\text{CDI}}_{\text{oral}/\text{dermal}}}{{\text{RfD}}_{\text{oral}/\text{dermal}}}, \end{array}$$

where *R*_*f*_*D* represents the reference dose (*μ*g·kg^−1^·day^−1^) equal to 40/12, 14/1.84, 300/60, 300/45, and 3/0.015 for oral and dermal exposure to Cu, Mn, Zn, Fe, and Cr, respectively.

#### 2.10.2. Hazard Index (HI)

The general potential noncarcinogenic effect of more than one element is explained by the sum of the calculated HQ of each metal, which is expressed by the HI. If HI is less than one (HI 1.0), no chronic risks are expected, but if HI exceeds one (HI > 1.0), a chronic risk is possible [25].

#### 2.10.3. Total Noncarcinogenic Risks (THI)

Considering that the risk is cumulative, the total noncarcinogenic risks associated with drinking water contamination are assessed using the THI, which is calculated as explained in the following equation [26]:

(7) $$\begin{array}{c} \text{THI}={\text{HI}}_{\text{oral}}+\text{HIdermal}. \end{array}$$

If the values are less than one (HI < 1), noncarcinogenic health effects are unlikely; however, if the values exceed one (HI > 1), noncarcinogenic health effects may occur [13].

### 2.11. Carcinogenic Risks

Cancer risk is defined as the risk of exposure to a lifetime average dose of 1 mg/kg body weight/day of a pollutant. It is measured in terms of incremental lifetime cancer risk (ILCR), which is the likelihood of developing cancer over 70 years as a result of 24 h of exposure to a potential carcinogen. The USEPA considers the acceptable cancer risk for regulatory purposes to be between 1 × 10^−6^ and 1 × 10^−4^ [25]. In this study, the ILCR was calculated for only the Cr element because no other metals with a possible carcinogenic risk were detected. Cancer risk was calculated as the product of CDI (mg/kg/day) and the cancer slope factor (CSF) (mg/kg/day^−1^), as explained in equation (8) [27]:

(8) $$\begin{array}{c} \text{ILCR}=\text{CDI}\times \text{CSF}, \end{array}$$

where ILCR = incremental life cancer risk; CDI = chronic intake (mg/kg/BW/day); and CSF = cancer slope factor. The CSFo value used for calculation was 5.0E^−1^ mg·kg^−1^·day^−1^.

### 2.12. Statistical Analysis

The collected data were subjected to analysis of variance (ANOVA) using SPSS software version 24.0 to assess the variation in heavy metal concentration between study sites. For each response variable, the validity of the model assumptions (normal distribution and constant variance assumptions in the error terms) was verified by examining the residuals. The mean separation was calculated using the least significance difference (LSD) at a probability level of 5% to generate letter groupings that would show a significant difference. The results obtained were presented using tables and graphs.

## 3. Results

### 3.1. Instrumental Calibration

The calibration curves were plotted to determine the concentration of heavy metals in the sample solutions. Calibration curves were plotted with six points for each standard metal solution using absorbance versus concentrations (mg/L). The values of the correlation coefficient (*R*^2^) values obtained ranged from 0.996 to 0.999, which is very close to the absolute value of 1, indicating that the parameters have a significant correlation (Table 5).

### 3.2. Level of Heavy Metals in Water Samples

The results of the heavy metal concentration from the river water samples are presented in Table 6. Copper, manganese, zinc, iron, and chromium were the heavy metals identified in the water samples, but Pb, Ni, Co, and Cd were not detected. Copper concentrations in the water samples ranged from 0.0560 to 0.4070 mg/L, with a mean of 0.19 ± 0.11 mg/L. A high copper content was recorded in Sam 2, followed by DK1, while the lowest concentration was observed in DK2. Manganese levels also ranged between 0.009 and 1.6990 mg/L, with a mean of 0.84 ± 0.65 mg/L. A high manganese concentration was recorded at the Yam 1 site, followed by the Mix 1 and Yam 2 sites, respectively, but the lowest concentration was observed at the DK1 and Sam1 sites. Zinc levels also ranged from 0.0037 to 0.5007 mg/L, with a mean of 0.18 ± 0.17 mg/L. A high concentration of Zn was measured at the Yam 1 Enz 1 and Yam 2 sites, respectively, while the lowest concentration was observed at the Sour 2 and Sour 1 sites. The highest and lowest iron concentrations were found in Yam 1 (27.85 mg/L) and DK1 (0.42 mg/L), with a mean of 10.31 ± 9.48 mg/L. Similarly, the Sam 2 and Yam 2 sites had the highest (0.17 mg/L) and lowest (0.02 mg/L) Chromium contents, with a mean of 0.10 ± 0.05 mg/L. The result showed significant heavy metal level concentration differences among the study sites for all metals analyzed metals (*p* < 0.05).

### 3.3. Multivariate Analysis of Heavy Metal Pollution Level and Study Sites

Table 7 shows the logarithmic relationship of principal component analysis (PCA) for the level of heavy metals pollution in river water and study sites. The relationship of parameters varied significantly on Axis 1 and Axis 2, accounting for 77.20% of the total variance.

**Figure 2:**
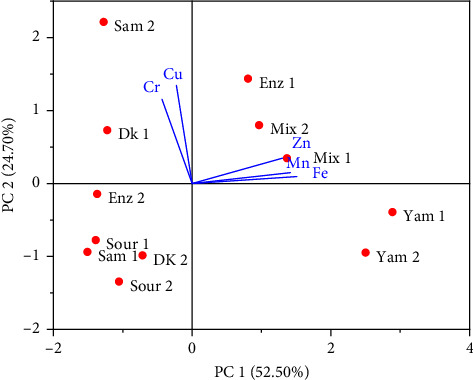
PCA analysis of the heavy metals' pollution level in the river water and study sites.

Mn, Zn, and Fe were positively correlated with the first axis with the highest Fe and Mn loads, whereas all heavy metals had a positive correlation on the second axis with the highest load contributed by Cu and Zn. On the other hand, the sites Yam 1, Yam 2, Mix 1, and Mix 2 contributed the highest positive on the first axis, while Sam 2, Enz 1, Mix 2, and Dk 1 contributed the highest positive on the second axis (Figure 2).

### 3.4. Pairwise Correlation of Heavy Metals

The pairwise correlation result of people also shows that Mn is positively correlated with Zn and Fe. Similarly, Fe showed a positive correlation with Mn and Zn, but Zn was positively correlated only with Fe. The correlations were strong and statistically significant (*p* < 0.05) (Table 8).

### 3.5. HPI and HEI

**Figure 3:**
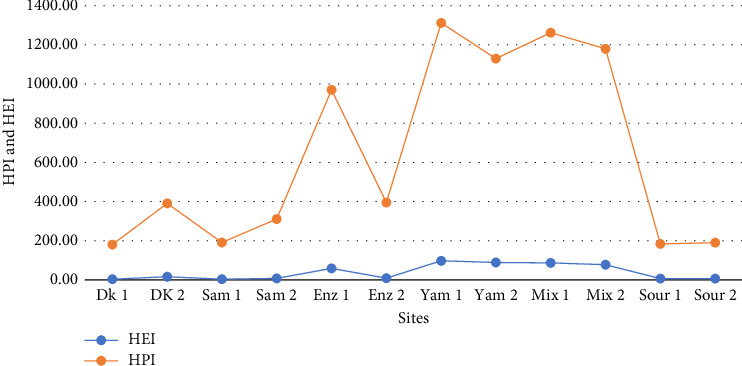
Heavy metal pollution index (HPI) and heavy metal evaluation index (HEI) among the different study sites.

The heavy metal pollution status is indicated by the cumulative concentrations of the metals studied (Cu, Mn, Zn, Fe, and Cr). HPI values ranged from 137.20 to 1277.79 with a mean of 118 ± 117.5. The highest and lowest values were recorded at the Yam 1 site and the Dk 1 site, respectively. The HEI values of the heavy metals evaluated also ranged from 3.24 to 99.31 with a mean of 39 ± 38.88, where the highest and lowest values were recorded at the Yam 1 and Dk 1 sites, respectively (Figure 3).

### 3.6. Chronic Daily Intake of Heavy Metals

Chronic noncarcinogenic risk after oral and dermal exposure was determined based on average concentrations of heavy metals in the water. Table 7 shows the CDI value computed for adults through oral ingestion and dermal contact pathways. Oral ingestion represented 99.55% and 97.85% of CDI_total_ (CDI_ingestion_ + CDI_dermal_ contact) in adults and children, respectively. On the other hand, the mean CDI values of all heavy metals were higher in children than in adults. The daily Fe and Mn intakes contributed to the majority of CDI_total_ in both target groups. The CDI_total_ contribution of heavy metals were in the order of Fe > Mn > Cu > Zn > Cr (Table 9).

### 3.7. HQ, HI, and THI

Mn showed the highest HQ_oral_ in both adults and children, followed by Fe and Cr. Similarly, Mn had the highest CDI_dermal_ in adults, followed by Fe and Cr. However, Cr experienced the highest HQ_dermal_ value in children, followed by Mn and Fe. Furthermore, Cr had HQ_oral_ value greater than a unit at most of the sites in children. In adults, HQ_dermal_ was less than one in all study sites, but in children, the Cr HQ_dermal_ of Cr was higher than one in Mix 1, Mix 2, and Sour 1.

Similarly, the HI_oral_ was greater than one at the study sites in both ages, except in DK 2, Sour 1, and Sour 2. In adults, HI_dermal_ was less than one except in Yam 1, Mix 1, Mix 2, and Sour 1. THI values were also higher than unity at all study locations in both ages, except for DK 2 and Sour 2 in adults and DK2 in children. In both ages, the noncarcinogenic risk of heavy metal was in the order of Mn > Fe > Cr > Cu > Zn (Tables 10 and 11).

### 3.8. Carcinogenic Risk Associated With Heavy Metal Consumption

ILCR examinations were only determined for Cr in this investigation. The Cr ILCR by ingestion of Koche River water was 0.75 ± 0.44 and 1.15 ± 0.66 for adults and children, respectively. The total CR obtained from Cr was 0.95 ± 0.58 (Table 12).

## 4. Discussion

### 4.1. Concentration of Heavy Metals in the Koche River

Heavy metal contamination is an important environmental problem in developing countries, due to uncontrolled levels of pollution caused by factors such as industrial expansion, excessive use of fertilizers and metal-based pesticides, industrial emissions, transportation, harvesting, storage, and/or sale [28, 29]. Cu, Mn, Zn, Fe, and Cr were the heavy metals identified in substantial amounts in the Koche River (Table 13). The studies indicate that heavy metals such as Cu, Mn, Zn, and Fe are essential heavy metals required for various biological processes in trace amounts, but if they exceed the threshold level they become toxic. On the other hand, hexavalent Cr is known for only its negative health and environmental impacts [35].

Copper is one of the essential micronutrients required for several biological processes including adequate growth, cardiovascular integrity, lung elasticity, neovascularization, neuroendocrine function, and iron metabolism [36]. However, it can cause several health problems including kidney damage, inhibition of urine production, and anemia due to rupture of red blood cells, when consumed in excess amounts [37]. The highest concentration of Cu (0.407 mL/L) observed at the SAMAKA 2 site of the current study could be attributed to the excessive use of fossil fuels and ores as energy sources in marble production. Studies also showed that a large amount of Cu (II) waste is generated by burning fossil fuels and ores in energy and metal-producing plants, as well as waste incinerators [38]. The results obtained in this study are within the threshold limit in drinking water (1.0 mg/L) [39]. It is also higher than Bedassa, Abebaw, and Desalegn [30] from Mojo River (0.004 mg/L), Meki River (0.009 mg/L), and Ziway Lake (0.003 mg/L), and G/Wold, Ayenew, and Ahmada [32] from Ogona River, Goba Town, but lower than Teklay and Amare [40] from Lake Hayq (0.26–1.940 mg/L). The variation could be attributed to differences in the amount of pollutants in the waste and the treatment methods used for discharges of wastewater into rivers.

Mn is another vital mineral that is necessary for healthy bone structure, reproduction, and central nervous system function [41]. However, long-term consumption of large amounts of manganese affects the central nervous system, visual reaction time, hand stability, and eye-hand coordination [42]. The highest level of Mn (1.69 mg/L) recorded at the Yam 1 site could also be related to the use of Mn input in food processing to enrich foods with manganese in Yamrot Food industry. Furthermore, manganese ore or metal is used in food production to produce various manganese salts [43]. The results of all sampling sites were higher than the recommended levels of WHO [39] (0.5 mg/L) and ESA [44] (0.1 mg/L), indicating that water is not suitable for drinking. It is also higher than the results of G/Wold, Ayenew, and Ahmada [32] in the Ogona River (0.094–0.123 mg/L) and Bedassa, Abebaw, and Desalegn [30] in the Mojo River (2.90 mg/L).

Zinc is also vital for the physiological and metabolic processes of organisms. Moreover, it is important for the growth and normal functioning of cells, protein synthesis, and carbohydrate metabolism [45]. However, higher levels of zinc consumption can cause system dysfunctions including growth impairment and reproduction, nausea, blood urine, liver failure, vomiting, epigastric pain, lethargy, and fatigue [46]. In this study, the use of zinc-rich inputs such as animal and plant ingredients in the food industry could have contributed the highest concentration of Zn (0.501 mg/L) in the water samples collected from the Yamrot 1 site [47]. The results of all sites were lower than the WHO [39] maximum Zn limit in drinking water (5 mg/L), but it is higher than the report by G/Wood, Ayenew, and Ahmada [32] (0.053–0.263 mg/L) on the Ogona River and Teklay and Amare [40] (0.150–0.160 mg/L) on Lake Hayq.

Iron (Fe) is also an essential micronutrient for hemoglobin formation, oxygen transport, and electron transport in the human body [48], but higher concentrations stored in the body through different routes cause health problems, such as organ failure and prolonged vomiting [49]. On the other hand, it causes the growth of iron-resistant bacteria in the water, causing water to acquire an unpleasant color, taste, and odor [50]. In our study, the water samples collected from Yamrot Food Complex factory (Yamrot 1) area contributes the highest amount of Fe (27.85 mg/L) in river water, indicating that agro-by-products could be the main source of Fe in the river water. Cámara et al. [51] also stated that iron content is high in the agro-by-products of the food industries because the percentage of dialyzable iron is low (less than 20%). Except for the SAMAKA 1 site, the water samples collected from all sites are not safe for drinking because the results recorded in these sites exceeded the WHO [39] (0.3 mg/L) and ESA [44] (0.3 mg/L) limit in drinking water. Our finding was also higher than in samples taken from Mekelle sources (0.097 mg/L) and the areas of Zalambessa (1.872 mg/L) [34] and the Ogona River (0.03–0.563 mg/L) [32].

The toxicity and carcinogenic properties of Cr (VI) have been reported with several health effects, such as ulcers, corrosive reactions in the nasal septum, acute irritative dermatitis, and allergic eczematous dermatitis [52]. In this study, the Sam 2 site exhibited the highest concentration of Cr (0.169 mg/L) in river water, which could be related to the incineration of coal or municipal waste for the generation of energy. It is used mainly to dehydrate and prevent corrosion. Similarly, Aukour and Al-Qinna [53] stated that the cement and stone industry is a major source of chromium. Except for samples collected at the source and Yam 2 sites, the recorded Cr content exceeded the WHO [39] maximum limit in drinking water (0.05 mg/L), indicating high a possibility of carcinogenic risk. It is also higher than the report by G/Wold, Ayenew, and Ahmada [32] (0.04–0.07 mg/L) in the Ogona River, but less than the report by Bedassa, Abebaw, and Desalegn [30] (2.039 mg/L) in the Mojo River, where the intensive leather industries discharge their wastes into the river. Generally, our result showed that heavy metals such as Mn, Zn, and Fe were positively correlated, indicating that these heavy metals have comparable or common potential sources [29].

### 4.2. HPI and HEI

HPI was used successfully in numerous investigations to assess heavy metal pollution in drinking water [11, 54]. In this study, many HPI values exceeded 100 in all water samples, indicating severe heavy metal contamination and unsuitability of the water for drinking. Our study confirmed a substantial disparity of HPI values most likely due to the high variability in Fe and Mn content in the study sites. This could be attributed to the volume of industrial waste and the concentration of heavy metals in the waste discharged into the river. This is consistent with the findings of Abebe et al. [8] at the majority of the study sites in the upper Awash River basin, with Zn, Fe, Cu, and Mn making significant contributions. Moyel et al. [55] also reported high HPI values attributed to high levels of Fe, Cd, Pb, and Ni in the water of the Shatt Al-Arab River, which exceeded the maximum allowable limit for drinking. Similarly, Dey et al. [56] reported that Cu and As are the main potential contributors to the high value of the pollution index in the Halda River water in Bangladesh. The recorded HPI values (137.20–1277.79) were comparable to the reports by Berego et al. [57] (0–436.09) in Hawassa Zuria, Boateng et al. [7] in hand-dug wells from Ejisu-Juaben Municipality, Ghana (319.20–688.05), and Moyel et al. [55] in Shatt Al-Arab River water (35–246).

Documented HEI (3.24–90.49) also indicates a high degree of river pollution (> 2) caused by heavy metal discharged from nearby industries. Similarly, studies conducted in Africa revealed moderate to severe heavy metal pollution in river water due to anthropogenic activities and lithogenic factors [1, 57]. Our finding is also consistent with Abebe et al. [8] (180–176.7), Moyel et al. [55] (30–200), Munene, Hashim, and Ambusso [58] (6.234–23.24), and Boateng et al. [7] (2.25–29.88).

### 4.3. Chronic Daily Intake of Heavy Metals

Exposure to heavy metals occurs primarily through drinking water, food, inhaled aerosol particles, and dust [59]. The toxicity level of heavy metals to human health is directly linked to the daily intake amount through various pathways [60]. Furthermore, long-term exposure to polluted water may pose health risks [55]. The finding of the present study indicates that dermal contact with river waters is less likely to be carcinogenic than oral ingestion, and this is consistent with several findings [8, 58, 61]. Due to the high concentrations of metals in the waste that enters the river at most of the study sites, iron (Fe) and manganese (Mn) contributed a higher noncarcinogenic risk than other metals through oral ingestion and dermal contact in both targeted groups. Nawab et al. [1] also reported the highest mean CDI values attributed to the highest Cr (3.61, 3.98 g/kg/day) and Zn (0.59, 0.65 g/kg/day) by oral ingestion from Buner District, northern part of Pakistan, for all ages. Similarly, Moldovan et al. [61] confirmed a significant contribution of Cr, Zn, and Ni to noncarcinogenic risks in some Karst water sources in Romania.

Our CDI value indicated that children are more vulnerable to heavy metal pollution and are more likely to experience adverse effects. This could be caused by the rapid development of the body, bone, and growth of children [62]. It could also be because adults have fully developed immune systems and are more likely to eliminate heavy metals [12]. Several studies have also found that children are more vulnerable to exposure to noncarcinogenic heavy metals than adults [8, 12, 57]. In general, the mean CDI values obtained from oral ingestion of all metals at both ages were higher than the RfD values, indicating a high noncarcinogenic risk. The CDI_oral_ values of the metals (1.50–161.97 *μ*g/kg/day and 2.30–247.67 *μ*g/kg/day in adults and children, respectively) were higher than the report of two sub-cities of Addis Ababa (0.05–1.08 *μ*g/kg/day for adults and 0.05–1.98 in children) [14] and the upper Awash Basin (3.8–14.1 *μ*g/kg/day for adults and 0.5–25 *μ*g/kg/day in children) [8].

### 4.4. HQ, HI, and Heavy Metal Total Hazard Index

The highest value of HQ_oral_ obtained above unit (> 1) for most metals at most study sites for both targeted groups corresponds to high concentrations of Mn, Fe, and Cr content at all sites, indicating an equal potential adverse health risk of long-term exposure. Similarly, Radfard et al. [13], Emmanuel et al. [5], and Khalid et al. [63] reported HQ above one in the water source of Fars Province, Iran; Anambra State, Nigeria; and Vehari, Pakistan, respectively, with a possible high health risk attributed to the high content of As, Cd, and Pb, respectively. In contrast, Niknejada et al. [64] reported an HQ of less than one for all heavy metals in Sari City, Iran, with a hierarchy of As > Cr > Mn > Pb > Cu > Fe > Zn. The difference could be attributed to heavy metal concentrations in water and pollution sources. The high level of HQ_oral_ and HQ_dermal_ in children than in adults implies that children are more vulnerable to noncancer risk, which is consistent with reports made in different areas [5, 61].

HI helps to estimate the general likelihood of noncarcinogenic effects posed by multiple elements [23]. In our study, the high HI_oral_ values (> 1) in both age groups in the majority of study areas (Yam 1, Yam 2, Mix 1, and Mix 2) signify the possibility of a noncarcinogenic oral impact on consumers. The highest HI_oral_ value could be attributed to the high Mn and Fe content detected in samples collected from these sites. On the contrary, HI values measured in drinking water sources from two Addis Ababa sub-cities [14] and Shenzhen, China [4] did not reveal noncarcinogenic risks from toxic heavy metals in both age groups. Children had higher levels of HI than adults, which could be attributed to shorter life spans and lower weight in children compared to teenagers and adults. A study in the upper Awash River basin [8], drinking water sources from the Akaki-Kality sub-city [14], and groundwater sources in Sari, Iran [9] also found a higher possibility of noncarcinogenic risk in children than in men.

The values of HI_oral_ (0.60–3.94 for adults and 0.029–5.409 for children) and HI_dermal_ (0.004–0.844 for adults and 0.029–5.409 for children) recorded in this study were higher than the report of Dessie et al. [14] from the two sub-cities of Addis Ababa water sources, who reported 2.31 × 10^−1^ and 4.20 × 10^−1^ through oral ingestion and 1.34 × 10^−1^ and 2.49 × 10^−1^ through dermal exposure in adults and children, respectively.

The highest THI (> 1) obtained at all study sites also indicates a possible noncarcinogenic risk for both adults and children through oral consumption. Similarly, Cr HQ_dermal_ greater than one recorded in Sour 1 and Sour 2 also indicates a high noncarcinogenic risk at these study sites. Dessie et al. [14] also reported THI_oral_ ranges of 0.134–4.20, which is comparable to our findings.

### 4.5. Carcinogenic Risk of Heavy Metals

According to the USEPA carcinogen reference dosage database, a risk level of 1 × 10^−4^ and 1 × 10^−6^ indicates 1 in 10,000 and 1,000,000 chances of developing cancer from drinking water containing elements such as Ni, Cr, Pb, and As in g/L for 70 years [65]. ELCR values less than one in a million (10^−6^) indicate a low probability of developing cancer, values between one in a million and one in 10,000 (10^−6^ to 10^−4^) are considered moderately risky, and values greater than one in 10,000 (10^−4^) are considered high risk [13]. The ELCR obtained in this study indicated a high carcinogenic risk of Cr (0.75 ± 0.44 for adults) and (1.15 ± 0.66 for children) if the community consumes river water. Similarly, studies found the possibility of Cr toxicity causing genotoxicity or cytotoxicity if the community consumed water in the upper Awash basin (0.0028 for adults and 0.0063 for children) [8] and Khorramabad, Iran [60] (mean ILCR 6.54 × 10^−3^ in adults).

The ILCR value recorded in this study was higher than in the upper Awash basin (0.0028 for adults and 0.0063 for children) [8], two Addis Ababa sub-cities (2.50 × 10^−4^ for adults and 4.5 × 10^−4^ for children) [14], and in the Mazandaran province, Iran (7.63 × 10^−5^ for adults 1.29 × 10^−4^ for children) [64]. The result showed that adults are more vulnerable to carcinogenicity risk than children, which agrees with the reports of Dessie et al. [14] and Niknejada et al. [64]. Therefore, efforts must be made to reduce the levels of pollution from pollutants and streams from nearby industries to prevent the occurrence of cancer in the future [6].

## 5. Conclusions

The present study confirmed that the water samples analyzed for the Koche River showed the presence of substantial toxic heavy metal pollutants (Cu, Zn, Cr, Fe, and Mn) in the river water due to the untreated wastewater that flows into the river water. Some of the detected metals (Cr, Fe, Mn, and Cu) exceeded the maximum limit for drinking water. Similarly, the computed HPI and HEI values indicate that the river is heavily polluted with heavy metals and is not suitable for drinking. However, oral ingestion of pollutants through drinking water is one of the main routes of heavy metal ingestion in which children are more vulnerable due to biological, physical, and occupational exposure. The HQ, HI, and THI recorded at most study sites also indicate the possibility of the development of noncarcinogenic risks by society in long-term exposure to these heavy metals. However, the result revealed an incremental development of lifetime cancer risk in 75 people out of 100 due to exposure to Cr content in river water. Therefore, proper control of wastewater discharged from industries is very critical to minimize its adverse impacts on the surrounding community. Finally, it is crucial to conduct similar studies on surface sediments, fish, and invertebrates to gain a more comprehensive understanding of heavy metal bioaccumulation and associated risks within the study area.

## Figures and Tables

**Table 1:** Study sites and their specific characteristics.

Site	Code	Northing	Easting	Specific characteristics
Site 1	Source 1	9° 1′ 27.0″	38° 37′32.2″	The source at which the river water originates
Site 2	Source 2	9° 1′ 27.4″	38° 37′32.6″	The site is 100 m from the river mouth
Site 3	DK 1	9° 1′ 27.3″	38° 37′49.6″	The site at the outlet of DK garment wastes
Site 4	DK 2	9° 1′ 28.9″	38° 37′59.2″	The location is 30 m after the DK1 sources join the river
Site 5	Enz 1	9° 1′ 35.6″	38° 37′48.5″	The site at the Ezana soap factory waste outlet
Site 6	Enz 2	9° 1′ 49.6″	38° 37′51.1″	The location is 30 m after the Enz 1 source joins the river
Site 7	Sam 1	9° 2′ 19.1″	38° 38′ 0.9″	The waste site at the Samaka marble factory outlet
Site 8	Sam 2	9° 1′ 55.5″	38° 38′15.5″	The location is 30 m after the Sam 1 sources join the river
Site 9	Yam 1	9° 1′ 56.2″	38° 38′17.2″	The site at the Yamirot food complex waste outlet
Site 10	Yam 2	9° 1′ 56.2″	38° 38′17.0″	The location is 30 m after the Yam 1 sources join the river
Site 11	Mix 1	9° 1′ 57.8″	38° 38′17.1″	The location at 50 m after all sources had mixed with the Koche River
Site 12	Mix 2	9° 1′ 55.3″	38° 38′17.4″	The second site at 100 m after mix 1

**Table 2:** The values of coefficients (*S*_*i*_, *I*_*i*_, guideline value, and *W*_*i*_) used in the HPI calculation.

Parameter	*S*_*n*_	*S*_*i*_ ∗ (*μ*g·L^−1^)	*I*_*i*_∗ (*μ*g·L^−1^)	*S*_*i*_ ppm	*W*_*i*_
Cu	500.00	1.50	0.05	1500.00	0.0007
Mn	300.00	0.30	0.10	300.00	0.0033
Zn	50.00	15.00	5.00	15,000.00	0.0001
Fe	2000.00	0.30	0.00	300.00	0.0033
Cr	3000.00	0.05	0.00	50.00	0.0200

*Note:* Source: [3, 21, 22].

**Table 3:** Parameters used to calculate the oral and dermal CDI of heavy metals.

No	Exposure parameter	Symbol	Units	Value	
				Adult	Children
1	Ingestion rate	IR	L/day	2.20	1.3.00
2	Exposure frequency	EF	Days/year	365.00	365.00
3	Exposure duration	ED	Years	70.00	70.00
4	Body weight	BW	kg	54.10	14.00
5	Average time	AT	Years	25,550	25,550.00
6	Exposed skin area	SA	cm^2^	18,000	7422.00
7	Exposure time	ET	h/day	25,550	25,550.00
8	Unit conversion factor	CF	L/cm^3^	0.001	0.001

*Note:* Source: [8, 14, 22, 25].

**Table 4:** Dermal permeability coefficient of heavy metals.

No	Metal	Oral RfD (mg/kg/day)	Dermal permeability coefficient (Kp) in cm/h
1	Mn	14	0.001
2	Fe	300	0.001
3	Cr	3	0.001
4	Cu	40	0.001
5	Zn	300	0.0006

*Note:* Source: [3, 22, 25].

**Table 5:** Calibration graph of absorbance versus heavy metal concentration in mg/L.

S.No	Metal	Model	*R* ^2^
1	Mn	*y* = 0.0880*x*	0.998
2	Fe	*y* = 0.0163*x*	0.998
3	Cr	*y* = 0.0477*x*	0.999
4	Cu	*y* = 0.0805*x*	0.996
5	Zn	*y* = 0.3320*x*	0.996

**Table 6:** The level of heavy metal concentration in water samples collected from different sites in the Koche River (in mg/L) (mean ± SE, LSD, CV, *n* = 3).

Site	Cu (mg/L)	Mn (mg/L)	Zn (mg/L)	Fe (mg/L)	Cr (mg/L)
Dk 1	0.340 ± 0.002^ab^	0.010 ± 0.002^f^	0.207 ± 0.01^bc^	0.424 ± 0.01^d^	0.078 ± 0.003^cd^
DK 2	0.056 ± 0.001^b^	0.796 ± 0.001^bc^	0.042 ± 0.001^c^	3.689 ± 0.24^d^	0.104 ± 0.003^bc^
Sam 1	0.063 ± 0.003^b^	0.109 ± 0.02^ef^	0.048 ± 0.001^c^	0.209 ± 0.001^d^	0.112 ± 0.004^abc^
Sam 2	0.407 ± 0.004^a^	0.719 ± 0.03^bc^	0.098 ± 0.003^c^	0.583 ± 0.002^d^	0.169 ± 0.020^a^
Enz 1	0.248 ± 0.01^ab^	0.892 ± 0.25^b^	0.408 ± 0.01^ab^	16.056 ± 0.12^c^	0.158 ± 0.004^ab^
Enz 2	0.098 ± 0.001^b^	0.379 ± 0.02^de^	0.052 ± 0.002^c^	1.396 ± 0.04^d^	0.156 ± 0.006^ab^
Yam 1	0.152 ± 0.003^ab^	1.699 ± 0.14^a^	0.501 ± 0.09^a^	27.845 ± 0.40^a^	0.035 ± 0.002^d^
Yam 2	0.115 ± 0.003^ab^	1.670 ± 0.15^a^	0.400 ± 0.001^ab^	25.322 ± 0.20^ab^	0.020 ± 0.002^d^
Mix 1	0.153 ± 0.004^ab^	1.685 ± 0.04^a^	0.175 ± 0.002^bc^	23.942 ± 0.16^ab^	0.131 ± 0.004^abc^
Mix 2	0.246 ± 0.01^ab^	1.521 ± 0.06^a^	0.149 ± 0.001^bc^	21.379 ± 0.26^bc^	0.121 ± 0.006^abc^
Sour 1	0.212 ± 0.001^ab^	0.154 ± 0.03^def^	0.019 ± 0.003^c^	1.464 ± 0.03^d^	0.040 ± 0.002^d^
Sour 2	0.151 ± 0.003^ab^	0.483 ± 0.02^cd^	0.004 ± 0.001^c^	1.376 ± 0.02^d^	0.025 ± 0.001^d^
LSD (0.05)	0.307	0.333	0.263	6.382	0.059

CV (%)	74.6	17.9	68.1	28.1	28.2

*p* value	0.038	0.0031	0.0013	0.0001	0.008

*Note:* n, no replication, the subscript ^abcdef^, the mean significant difference between sample sites: DK1, DK garment factory outlet site; DK2, the second sample of DK garment factory; Sam 1 sample at the outlet of the SAMAKA STONE marble factory; Sam second sample site of the SAMAKA STONE marble factory; Enz 1Enzat soap factory outlet; Enz 2 s sample at Enz soap factory; Yam 1, Yamrot food complex outlet; Yam 2, second Yamrot food complex outlet; Mix1, the first site at which all sources mixed with Koche River; Mix 2, the second site after mixed Koche River; Source 1, sample from Koche River sources; Source 2, the second sample of Koche sources at 100 m from the source.

Abbreviation: CV, coefficient of variation.

**Table 7:** Percentage of variance heavy metals pollution level in the river water and study sites on the first and second axes.

Heavy metal	PC1	PC2
Eigenvalue	2.62485	1.23514
Percentage of variance (%)	52.50	24.70
Cumulative (%)	52.50	77.20
Cu	−0.09066	0.74141
Mn	0.5682	0.08174
Zn	0.52215	0.19142
Fe	0.60491	0.05225
Cr	−0.17424	0.63582

**Table 8:** Pairwise comparison of detected heavy metals in the Koche River.

Metal	Cu	Mn	Zn	Fe	Cr
Cu	1	−0.124	0.095	−0.131	0.272
Mn	−0.124	1	0.626⁣^∗^	0.941⁣^∗∗^	−0.091
Zn	0.095	0.626	1	0.775⁣^∗^	−0.184
Fe	−0.131	0.941⁣^∗∗^	0.775⁣^∗^	1	−0.182
Cr	0.272	−0.091	−0.184	−0.1821	1

^∗^ and ^∗∗^indicate a significant difference at 95 and 99% CL, respectively.

**Table 9:** Chronic daily intake (CDI) of heavy metals by adults and children in the study area.

Age	Route	Metal	Min	Max	Mean	STD	% Contribution
Adult	Oral	Cu	0.88	6.40	2.93	1.70	1.59
		Mn	0.14	26.70	13.25	10.23	7.23
		Zn	0.06	7.87	2.75	2.49	1.50
		Fe	3.28	437.56	161.97	150.42	88.34
		Cr	0.31	2.66	1.50	0.86	0.82
	Dermal	Cu	0.01	0.03	0.02	0.01	0.01
		Mn	0.001	0.14	0.07	0.05	0.04
		Zn	0.00	0.03	0.01	0.004	0.01
		Fe	0.02	2.30	0.85	0.65	0.47
		Cr	0.002	0.05	0.01	0.003	0.004

Children	Oral	Cu	1.35	9.78	4.49	2.60	1.58
		Mn	0.22	40.83	20.26	15.64	7.11
		Zn	0.09	12.03	4.21	4.01	1.48
		Fe	5.02	669.10	247.67	225.88	86.89
		Cr	0.31	4.06	2.30	1.31	0.81
	Dermal	Cu	0.03	0.22	0.10	0.06	0.03
		Mn	0.01	0.90	0.45	0.35	0.16
		Zn	0.001	0.16	0.06	0.05	0.02
		Fe	0.11	14.76	5.46	5.09	1.92
		Cr	0.01	0.09	0.05	0.03	0.02

**Table 10:** Oral and dermal hazard quotient (HQ), HI, and THI of heavy metals in adults.

Metal	Oral						Dermal						THI
	Cu	Mn	Zn	Fe	Cr	ΣHQ = HI	Cu	Mn	Zn	Fe	Cr	ΣHQ = HI	
Dk 1	0.13	0.89	0.01	0.19	0.41	**1.64**	0.002	0.036	0.000	0.007	0.002	**0.047**	**1.69**
DK 2	0.02	0.01	0.00	0.02	0.54	**0.60**	0.000	0.000	0.000	0.001	0.003	**0.004**	**0.61**
Sam 1	0.16	0.81	0.01	0.03	0.59	**1.59**	0.003	0.032	0.000	0.001	0.003	**0.039**	**1.63**
Sam 2	0.02	0.12	0.00	0.01	0.89	**1.05**	0.000	0.005	0.000	0.000	0.005	**0.010**	**1.06**
Enz 1	0.10	1.00	0.02	0.84	0.83	**2.79**	0.002	0.040	0.000	0.030	0.004	**0.076**	**2.86**
Enz 2	0.04	0.43	0.00	0.07	0.82	**1.36**	0.001	0.017	0.000	0.003	0.004	**0.025**	**1.38**
Yam 1	0.06	1.91	0.03	1.46	0.18	**3.63**	0.001	0.076	0.000	0.051	0.001	**0.130**	**3.76**
Yam 2	0.05	1.87	0.02	1.33	0.10	**3.37**	0.001	0.075	0.000	0.047	0.110	**0.233**	**3.60**
Mix 1	0.10	1.89	0.01	1.25	0.69	**3.94**	0.002	0.076	0.000	0.044	0.722	**0.844**	**4.78**
Mix 2	0.06	1.71	0.01	1.12	0.63	**3.53**	0.001	0.068	0.000	0.039	0.664	**0.773**	**4.30**
Sour 1	0.08	0.54	0.00	0.08	0.21	**0.91**	0.001	0.022	0.000	0.003	0.221	**0.246**	**1.16**
Sour 2	0.06	0.17	0.00	0.07	0.13	**0.44**	0.001	0.007	0.000	0.003	0.138	**0.148**	**0.58**

*Note:* The bold results represent the hazard index (HI) of each heavy metal across the study areas.

**Table 11:** Oral and dermal hazard quotient (HQ) of heavy metals in children.

Metal	Oral						Dermal						THI
	Cu	Mn	Zn	Fe	Cr	ΣHQ = HI	Cu	Mn	Zn	Fe	Cr	ΣHQ = HI	
Dk 1	0.20	1.37	0.02	0.30	0.62	**2.51**	0.015	0.229	0.001	0.043	0.014	**0.303**	**2.81**
DK 2	0.03	0.02	0.00	0.03	0.83	**0.92**	0.002	0.003	0.000	0.005	0.018	**0.029**	**0.95**
Sam 1	0.24	1.23	0.01	0.05	1.35	**2.88**	0.018	0.207	0.001	0.007	0.020	**0.252**	**3.13**
Sam 2	0.04	0.19	0.00	0.02	0.90	**1.15**	0.003	0.031	0.000	0.002	0.030	**0.067**	**1.22**
Enz 1	0.15	1.53	0.03	1.29	1.27	**4.26**	0.011	0.257	0.002	0.189	0.028	**0.487**	**4.75**
Enz 2	0.06	0.65	0.00	0.11	1.25	**2.07**	0.004	0.109	0.000	0.016	0.028	**0.158**	**2.23**
Yam 1	0.09	2.92	0.04	2.23	0.28	**5.56**	0.007	0.490	0.003	0.328	0.707	**1.52**	**7.10**
Yam 2	0.07	2.87	0.03	2.03	0.16	**5.16**	0.005	0.481	0.002	0.298	0.006	**0.79**	**5.95**
Mix 1	0.15	2.89	0.01	1.92	1.05	**6.02**	0.011	0.485	0.001	0.282	4.630	**5.409**	**11.43**
Mix 2	0.09	2.61	0.01	1.71	0.97	**5.39**	0.007	0.438	0.001	0.252	4.259	**4.956**	**10.35**
Sour 1	0.13	0.83	0.00	0.12	0.32	**1.40**	0.009	0.139	0.000	0.017	1.414	**1.580**	**2.98**
Sour 2	0.09	0.26	0.00	0.11	0.20	**0.67**	0.007	0.044	0.000	0.016	0.884	**0.951**	**1.62**

*Note:* The bold results represent the hazard index (HI) of each heavy metal across the study areas.

**Table 12:** The carcinogenic risk associated with Cr metal consumption in the study area.

Variable	Min.	Max.	Mean ± SD
CDI adult	0.31	2.66	1.50 ± 0.86
CDI children	0.48	4.06	2.30 ± 1.31
CDI total	0.31	4.06	1.90 ± 1.16
CSFo	0.50	0.50	0.50 ± 0.00
CR adult	0.16	1.33	0.75 ± 0.44
CR children	0.24	2.03	1.15 ± 0.66
CR total	0.16	2.03	0.95 ± 0.58

**Table 13:** The mean concentrations of heavy metals (mg L^−1^) in water samples of Koche River and international standard values (WHO (1984), ESA (2013) and in other similar rivers.

No	Heavy metal	Concentration in the current river (mg/L)	WHO (1984) limit	ESA (2013) limit	Concentration in other rivers (mg/L)	References
1	Cu	0.19 ± 0.11	1.00		Mojo river (0.004)	[30]
					Meki river (0.009)	[30]
					Golli river (2.148 ± 0.236)	[31]

2	Mn	0.84 ± 0.65	0.50	0.1	Ogona river (0.094–0.123)	[32]
					Mojo river (2.90)	[30]
					Megech river (0.01–0.02)	[33]

3	Zn	0.18 ± 0.17	5.00		Ogona river (0.053–0.263)	[32]
					Golli river (0.9725 ± 0.89)	[31]
					Megech river (0.11–0.16)	[33]

4	Fe	10.31 ± 9.48	0.30	0.3	Mekelle water sources (0.097)	[34]
					Ogona river (0.03–0.563)	[32]
					Megech river (2.5–7.6)	[33]

5	Cr	0.10 ± 0.05	0.05		Ogona river (0.04–0.07)	[32]
					Mojo river (2.039)	[30]
					Golli river (0.167 ± 0.15)	[31]

## Data Availability

The majority of data are already illustrated in the result part. The raw data of this work would be available upon reasonable request.
